# Locomotor activity in males of *Aedes aegypti* can shift in response to females’ presence

**DOI:** 10.1186/s13071-018-2635-9

**Published:** 2018-04-18

**Authors:** Luciana Ordunha Araripe, Jéssica Rodrigues Assunção Bezerra, Gustavo Bueno da Silva Rivas, Rafaela Vieira Bruno

**Affiliations:** 1 Laboratório de Biologia Molecular de Insetos, Instituto Oswaldo Cruz, FIOCRUZ, Rio de Janeiro, Brazil; 2 Department of Entomology and Nematology, Citrus Research and Education Center, University of Florida, Lake Alfred, FL USA; 3 Instituto Nacional de Ciência e Tecnologia em Entomologia Molecular/CNPq, Rio de Janeiro, Brazil

**Keywords:** *Aedes aegypti*, Reproductive behavior, Mate recognition, Mosquitoes, Circadian activity, Vector control

## Abstract

**Background:**

The study of physiological and behavioral traits of mosquito vectors has been of growing relevance for the proposition of alternative methods for controlling vector-borne diseases. Despite this, most studies focus on the female’s traits, including the behavior of host seeking, the physiology of disease transmission and the site-choice for oviposition. However, understanding the factors that lead to males’ reproductive success is of utmost importance, since it can help building new strategies for constraining population growth. Male behavior towards mating varies widely among species and the communication between males and females is the first aspect securing a successful encounter. Here we used an automated monitoring system to study the profile of locomotor activity of *Aedes aegypti* males in response to female’s presence in an adapted confinement tube. We propose a new method to quantify male response to the presence of females, which can be potentially tested as an indicator of the success of one male in recognizing a female for mating.

**Results:**

Locomotor activity varies in daily cycles regulated by an endogenous clock and synchronized by external factors, such as light and temperature. Our results show the previously described startle response to light, which is displayed as a steep morning activity peak immediately when lights are on. Activity drops during the day and begins to rise again right before evening, happening about 1.5 h earlier in males than in females. Most interestingly, males’ activity shows a double peak, and the second peak is very subtle when males are alone and relatively more pronounced when females are present in the confinement tubes. The switch in the peak of activity, measured by the herein suggested Peak Matching Index (PMI), was significantly different between males with and without females.

**Conclusions:**

The adapted monitoring system used here allowed us to quantify the response of individual males to nearby females in terms of the extent of the activity peak displacement. In this direction, we created the peak matching index (PMI), a new parameter that we anticipate could be interpreted as the inclination of males to respond to females’ presence, and further tested as an indicator of the potential for finding females for mating.

## Background

The study of male behavioral and physiological traits has been historically neglected in species of blood-sucking mosquitoes. This is mostly because males do not bite and are not vectors of diseases themselves [1–4]. Nevertheless, male reproductive behavior has a primary role on the growth and perpetuation of mosquito populations, as any factor found to lead to reproductive failure will discontinue population growth. Moreover, males in copula produce and transfer seminal substances from their accessory glands that are able to modulate the behavior of females, and some of these behavioral changes may influence the female’s vectorial capacity [5–7]. Therefore, the study of male behavior towards mating has an enormous potential of contributing to existing vector control methods based on the release of genetically modified males [8–11].

The mosquito *Aedes aegypti* is one of the main vectors of arboviruses in tropical areas. In addition to being the major vector of yellow fever, dengue, chikungunya and Zika, its populations are increasing and its distribution is expanding to subtropical areas, making the need for strategies of vector control extremely urgent. Due to the increase of insecticide resistance [12–16], the World Health Organization (WHO) encourages the application of methods that go beyond the use of insecticides and target the fertility of adults and the viability of eggs and larvae. These methods benefit from the knowledge of the insect vector’s reproduction and embryology and must have the potential of reducing population growth. Besides avoiding environmental contamination with insecticides, the major advantage of methods targeting reproduction is that they rely on the natural specificity of recognition and the encounter of males and females from one species [3, 8, 17]. Thus, it is of great interest to study the factors that mediate the communication between males and females before the mating event, and knowledge is currently lacking for *Ae. aegypti* and other insect vectors [18].

While reproductive behaviors have a recognized genetic basis, certain aspects of these behaviors are influenced by social experience and may show plastic variation [19, 20]. In *Drosophila*, it was shown that males that have experienced contact with same-sex or opposite sex individuals do perform differently in later encounters [19, 21, 22]. The olfactory system of males performs the major role in detecting volatile pheromone signals in fruit flies, but non-volatile pheromones can also be detected by the gustatory system and work on stimulating the initiation of the courtship ritual [19, 23, 24].

Although well characterized in *Drosophila*, the role of volatile pheromone signals in mosquito reproduction is still questioned [4, 25, 26]. Equipped with an extremely sensitive hearing organ, the antenna, it is believed that males of most species of mosquitoes respond mainly to acoustic cues for recognizing and pursuing mating with conspecific females [4, 27–30]. It was recently shown that the flight towards mating involves harmonic convergence, which means that males and females mutually convert their wing-beat frequencies up to a matching frequency [11, 31]. Nevertheless, even though the influence of volatile pheromones in the communication of *Ae. aegypti* has been considered as minimal or absent, it has been reported that both swarming males and nearby females produce volatile pheromones that function as stimulators of the flying activity towards the swarm [32].

Locomotor activity in insects is a manifestation of a collection of behaviors associated with basic functions like foraging, mating, host seeking and oviposition. Most organisms, including mosquitoes, experience these functions in cycles, depicting daily rhythms that are characteristic of the species [25, 33, 34]. Some of these rhythmic features withstand even when individuals are submitted to conditions of constant darkness and stable temperature, revealing that rhythmic functions are under the regulation of an endogenous circuit called the circadian clock [35–37]. While regulation happens in the intracellular level, environmental conditions like light, temperature, food availability and substrate vibration are the major synchronizers of the clock [38–42], entraining the endogenous loops of gene regulation so that the rhythmic behavior happens every 24 h. In chronobiology, environmental synchronizers such as light and temperature, among others, are called *Zeitgebers*, and are partly responsible for the variation in locomotor activity observed within and between species.

The study of locomotor activity has progressed in a number of insect species, especially with the use of acoustic or photoelectric actographs [43, 44]. The use of automated monitoring systems offers important benefits, like the possibility of measuring individual activity, in short time intervals, across several days in controlled laboratory conditions. This is advantageous because it eliminates the need for one or more observers, which is laborious and may introduce subjectivity.

Here we studied in laboratory conditions the locomotor/flight activity of *Ae. aegypti* males in response to female’s presence at a certain distance or female’s absence. Furthermore, we propose a new method to identify and quantify shifts in a male’s activity profile. The proposed index herein measured the shift resulting from the perception of a female’s presence, and thus we anticipate that it can be potentially used to address the success of one male in recognizing and finding a female for mating.

## Methods

### Mosquito rearing

All experiments were carried out with mosquitoes from laboratory colonies of *Ae. aegypti* (Rockefeller strain), maintained by the Laboratório de Fisiologia e Controle de Artrópodes Vetores (LAFICAVE). Mosquitoes were synchronized from larvae to adults, to 12 h of light and 12 h of darkness (LD) under a constant temperature of 25 °C. Virgin males and females were separated, beginning a few hours after emerging from pupae, and the cage was checked twice a day, in order to guarantee that newly emerged adults would not mate. After obtaining the necessary number of virgin females and males in separate cages, we let the following emerged adults mate freely in the original cage, so we could use a set of inseminated females for the experiments. Adults were removed from cages with an automatic insect aspirator and anesthetized on ice, until placed in experimental tubes.

### Activity monitors and adapted confinement system

In order to measure the circadian profile of locomotor activity in *Ae. aegypti*, we used activity monitors (Trikinetics LAM25, Waltham, MA, USA) to record the number of movements per unit of time. As most of the locomotor activity of *Ae. aegypti* is manifested as flight, and flight (wing-beat frequency) is very important for the recognition between males and females of the same species, we chose to use a monitoring system that can house tubes large enough to allow individuals to take flight. The equipment used was an infrared movement detection system, built as an acrylic vertical panel with 32 infrared emission/capture rings. Glass cylindrical tubes (25 × 150 mm) with one insect each were placed across each infrared ring, and when the insect crossed the infrared, one unit of movement was recorded. The daily locomotion was recorded in 5 min intervals using the DAMSystem3 Software. For representation and analysis, data were transformed to 30 min intervals by summing each six recordings of 5 min. By plotting all movements recorded over several days it was possible to identify daily locomotor/flight activity rhythms for each individual tested.

**Fig. 1:**
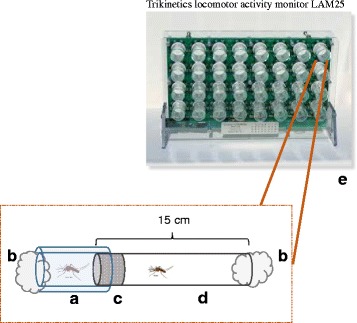
Graphical scheme of the adapted confinement tube. **a** Plastic tube used as confinement for one female. **b** Cotton embedded in 10% sacarose solution. **c** Piece of tulle net used to separate male from female. **d** Glass cylinder placed in one of 32 rings of the activity monitor. **e** Each tube mounted is lodged in one of the monitor rings

The advantage of using the LAM25 system is that the individual activity of up to 160 males can be recorded (using five identical monitors), in short intervals of less than 1 min, over several days inside an incubator. This allows for parameters of the circadian activity be precisely estimated in specific conditions of temperature and photoperiod. Taking advantage of this system, where each individual is placed inside a glass cylindrical tube with a food source provided in one end, we created a manner of measuring the locomotor/flight activity likely related to the perception of females by males. We adapted plastic tubes (26 × 50 mm) to one end of the glass tubes and made the two environments separate by a tulle net (Fig. 1). The plastic tubes were called confinement tubes. Females were placed individually in the confinement tubes and males were placed in the glass tubes. The net allowed for males and females to communicate acoustically, visually and chemically, while it impeded mating. Figure 1 shows a graphical scheme of the adapted confinement tube and a picture of the activity monitor lodging the experiment tubes.

### Test of confinement tubes

Before initiating our analyses of locomotor activity, we decided to test the adapted confinement tubes with a pilot experiment (Exp1). We used three different treatments: (i) males in glass tubes, with no confinement tubes attached; (ii) males in glass tubes and empty (no female) confinement tubes; and (iii) males in glass tubes with virgin females confined in plastic tubes. All tubes were provided with a food source: a piece of cotton embedded in 10% sucrose solution. Both males and females were kept well and alive inside the tubes for the length of the experiment (15 days). Moreover, dissection of females showed that the tulle net prevented mating in 100% of the cases. The female’s three spermathecae were dissected using a pair of forceps on a microscopy slide, and visually inspected under the microscope for the presence of sperms. All females previously classified as virgins had their spermathecae empty.

### Experimental design

After Exp1, four separate 15 day experiments were performed, in which mosquitoes were maintained for 4 days in a LD cycle (12 h light/12 h dark) and 11 days in a DD regime (constant darkness). Across these experiments we not only used different conditions in the plastic tubes, but also replicated conditions and replicated controls, in order to increase sample size and verify repeatability of results at different time points. In each of Exp2, Exp3 and Exp4, we measured the locomotor/flight activity of 160 males, separately, in four different treatments: (i) with virgin females confined in the plastic tubes, providing a food source (cotton embedded in 10% sucrose solution) in both tubes; (ii) with inseminated females confined in the plastic tubes, providing a food source in both tubes; (iii) without females in the plastic tubes, but yet providing a food source in both tubes, to eliminate the possibility that the male could be reacting to the presence of a food source instead of reacting to the presence of a female; and in (iv) we measured the activity of females in glass vials, without males confined (replicated only in Exp3 and Exp4). All experiments were run in the same incubator (Precision Scientific 818, Chennai, India), set to 25 °C.

In order to understand the nature of the communication signals between males in glass tubes and females in confinement tubes, we performed Exp5 using individuals with ablated organs. Again, we used four different treatments: (i) males with ablated antennae, with virgin females confined in plastic tubes; (ii) males with ablated antennae, with no females in confinement tubes; (iii) males not ablated, with virgin females wing-ablated in confinement tubes; and (iv) males and virgin females not ablated as a control. For these experiments, all females were virgin. The ablated-mosquitoes experiment was also performed for 4 days in a LD cycle (12 h light/12 h dark) and 11 days in a DD regime (constant darkness).

### Data analysis

Locomotor activity data were analyzed using the software Actogram J [45]. All activity data recorded were summed up in intervals of 30 min and averaged among individuals across each 30 min interval using the William’s mean [46, 47]. The William’s mean consists of calculating the geometric mean instead of a regular arithmetic mean. Since the mosquito activity data are especially variable, transforming the data to logarithm allows their distribution to be more constrained and the average to be less influenced by very low or very high values. In fact, because we have many zeros in the data series, we must use log (*n* + 1) instead of log *n*. The advantage of using this calculation is that it prevents the masking of data by the effect of very high numbers within a single interval.

The William’s mean per interval of 30 min was first calculated across individuals for every day of experiment, and then averaged across the same intervals of days 2, 3 and 4 of the LD cycle (12 h light/12 h dark). This result was used to graphically represent the average profile of activity within a period of 24 h, of males tested with or without the presence of females in the confinement tubes, as well as the females’ profile of activity. The first day of LD cycle was regarded as a period when mosquitoes were adapting to the confinement tubes. Thus, we chose not to include data from day 1 in the calculation of the average profile.

Data generated during 4 days in LD cycle and 11 days in DD cycle were analyzed separately. The total activity in LD and DD cycles, the proportion of activity in DD, as well as parameters of the individual activity profiles, like the magnitude of activity at the peak and the phase of the peak, were compared among treatments using analysis of variance (ANOVA) with Tukey’s *post-hoc* test. Where the variances were not homoscedastic according to the Bartlett’s test, one of three transformations was applied to the data (log, square root or arcsine) before using ANOVA. When data transformation did not provide homoscedastic variances, comparisons relied on the non-parametric Kruskal-Wallis with Dunn’s multiple comparisons test. Statistical tests were performed with the use of the software GraphPad Prism (Prism, La Jolla, CA, USA) and the R package.

As detailed in the Results section, the activity profile of males presented two well-defined evening peaks, which were called E1 and E2. For the purpose of statistical comparisons among treatments, individual activity profiles were used: we calculated the William’s mean across the same 30 min intervals of days 2, 3 and 4 for each individual, and identified the peaks of activity from each individual’s average profile. The phases of peaks E1 and E2 could be identified between ZT8.5 to ZT12 (from 8.5 h till 12 h after the *Zeitgeber* light was on). Besides comparing the magnitude of each of these peaks among treatments, we created an index to measure the relationship between the two peaks with one single value. This index was called peak matching index (PMI) and was calculated as the magnitude of peak E2 minus the magnitude of peak E1, divided by the average of the two peaks. A positive value of PMI indicates that the second peak of activity (E2) is greater than the first (E1), which means that the main activity appears as a second evening peak. PMI was calculated individually, e.g. using the first and second peaks of each male. The Bartlett test for homoscedasticity of variances was used over parameters E1, E2 and PMI and, where the test was significant, log transformation was applied before running an analysis of variance (ANOVA) with Tukey’s *post-hoc* test.

Data generated when individuals were in the DD regime were used to calculate the period length and the power of activity. In order to calculate these parameters, individuals are monitored for at least 10 days in free running condition (constant darkness), recommended as a regular procedure when studying the circadian rhythms of activity in insects [25, 48–50]. Light/dark cycles are responsible for synchronizing the endogenous clock, which means that in a LD cycle, the peak(s) of activity will happen every day at the same time. However, when the *Zeitgeber* light is absent and individuals are submitted to constant darkness (DD), the endogenous rhythm is revealed and the period length may be shorter or longer than 24 h. It was previously found that the period length is shorter than 24 h (about 22 h) in *Ae. aegypti* [49, 51], which means that the endogenous regulation of clock genes is cycling every 22 h, regardless of an environmental cycling condition. Our design allowed the same individuals be studied in LD and DD.

The free-running period length (tau) of each individual was calculated automatically, using the Lomb-Scargle periodogram adjustment in the software ActogramJ, which is based on Fourier analysis [45]. The strength of the rhythm was estimated using the parameter power [52], which is given by the distance from the peak of the periodogram to the confidence level in the Lomb-Scargle adjustment. The parameters period length and power were compared among treatments using the non-parametric Kruskal-Wallis with Dunn’s multiple comparisons test.

## Results

**Fig. 2:**
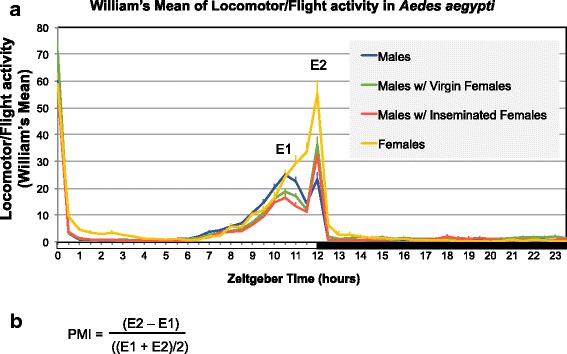
**a** Average profile of the locomotor/flight activity of *Ae. aegypti* males in different treatments, across 24 h. Average refers only to days 2, 3 and 4 in LD (12 h light/12 h dark). The first day in LD was not included because we considered males to be still adapting to the system. Error bars are shown for each 30 min interval. X-axis: *Zeitgeber* time refers to the number of hours after the light turns on inside the incubator; the white bar represents 12 h of light and the black bar represents 12 h of darkness. **b** Formula used for calculating the peak matching index (PMI)

Our results show that both males and females of *Ae. aegypti* mosquitoes have a startle response to light, producing a steep morning activity peak at ZT0 (*Zeitgeber* 0), immediately when lights are on. This response to light was observed in previous works, where experiments in free running conditions (constant darkness) have shown that this activity is not present [49, 53, 54]. Locomotor activity drops steeply at ZT1 (1 h after the *Zeitgeber* light is on) and keeps low or null until ZT7, when it begins to rise, reaching a peak at ZT10.5 in males (without females) and a peak at ZT12 in females (without males) (Fig. 2). The early peak of activity in males in relation to females had been previously reported [49, 55], and although the reason was not investigated in details, it could be speculated as a manifestation of male swarming activity happening before the time females are the most active. Most interestingly, locomotor activity shows a double peak in males and the second peak (E2) is very subtle when males are alone (mean ± SD, 29.9 ± 25.5), and significantly more pronounced (39.5 ± 31) when virgin females are present in the confinement tubes (*F*_(3, 331)_ = 13.68, *P* < 0.0001, Tukey’s test: *P* = 0.049) (Fig. 2, Table 1). This indicates that the presence of females is not only noticed by males, but males delay their main activity peak to overlap with the peak of females, implying they are capable of changing their pattern of activity in response to females.

**Table 1:** Statistical comparisons of locomotor-activity parameters E1 (evening peak of activity 1), E2 (evening peak of activity 2) and PMI (peak matching index). ANOVA with Tukey’s *post-hoc* test used to compare parameters among treatments. Results of pairwise comparisons for each parameter are shown as superscript letters; in each column the different letters represent significant difference among treatments

Treatment	*n*	E1	E2	PMI
		Mean ± SD	Mean ± SD	Mean ± SD
Males	94	29.8 ± 19.3^a^	29.9 ± 25.5^a^	-0.1 ± 0.7^a^
Males_VF	100	24.9 ± 13.8^ab^	39.5 ± 31.0^b^	0.3 ± 0.7^b^
Males_IF	69	21.7 ± 14.6^b^	34.7 ± 27.9^ab^	0.3 ± 0.7^b^
Females	72	20.6 ± 11.6^b^	63.6 ± 42.1^c^	0.9 ± 0.6^c^
ANOVA *F* (*P*)		5.019 (0.002)	13.68 (< 0.0001)	31.47 (< 0.0001)

*Abbreviations*: *M_VF* males with virgin females, *M_IF* males with inseminated females

As described above, in order to measure the shift in the evening activity peaks with one single value, we created the peak matching index (PMI). The PMI made it possible to characterize the relationship between the two peaks of activity and to make this relationship comparable among treatments. The PMI is defined in the Methods section and shown in Fig. 2.

**Fig. 3:**
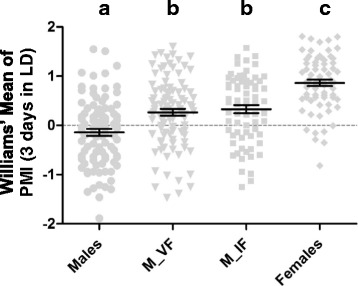
Plot of PMI values for each treatment, with mean and standard errors represented by innermost and outer bars, respectively. Comparison among treatments performed using ANOVA with Tukey’s *post-hoc* pairwise tests shown as lowercase letters. *Abbreviations*: M_VF, males with virgin females; M_IF, males with inseminated females

**Fig. 4:**
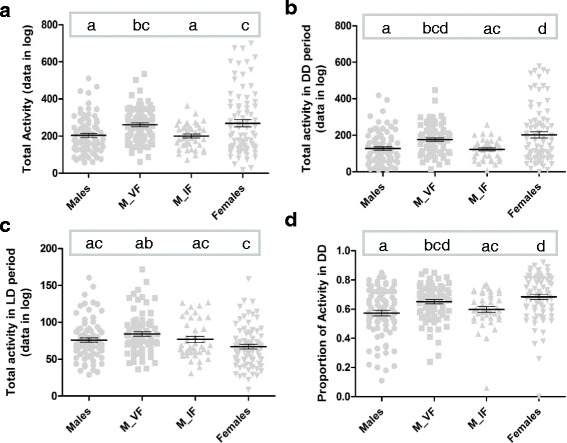
Plot of total activity parameters for each treatment, with mean and standard errors represented by innermost and outer bars, respectively. Comparisons for the total activity in LD (graph **c**) were performed using ANOVA, with Tukey’s *post-hoc* pairwise tests shown as lowercase letters. The parameters total activity (**a**), total activity in DD (**b**) and proportion of activity in DD (**d**) were compared among treatments with the non-parametric Kruskal-Wallis test, with Dunn’s multiple comparisons test shown as lowercase letters. *Abbreviations*: M_VF, males with virgin females; M_IF, males with inseminated females

In all experiments, PMI was significantly different between males with (0.3 ± 0.7) and without females (-0.1 ± 0.7) confined in plastic tubes (*F*_(3, 331)_ = 31.47, *P* < 0.0001, Tukey’s test: *P* = 0.00014) (Fig. 3, Table 1). This result shows that male activity behavior in *Ae. aegypti* is significantly altered by the presence of one female nearby, suggesting that male’s circadian rhythm can be manipulated by the perception of signals emitted by females. Not only does the shape of the activity profile change when females are present, the total activity in 24 h (*χ*^2^ = 18.06, *df* = 3, *P* = 0.0004, Dunn’s test: *P* = 0.0001) and the total activity in DD (*χ*^2^ = 20.96, *df* = 3, *P* = 0.0001, Dunn’s test: *P* < 0.0001) were significantly different between males with no female and males with a virgin female in the confinement tube (Fig. 4 and Table 2). Interestingly, for both LD and DD regimes, the total activity of males without females was not significantly different from the total activity of males with inseminated females in the confinement tubes, and only the parameter of total activity in 24 h differed significantly between males with virgin females and males with inseminated females (*χ*^2^ = 18.06, *df* = 3, *P* = 0.0004, Dunn’s test: *P* = 0.0013) (Fig. 4 and Table 2).

**Table 2:** Statistical comparisons of locomotor-activity parameters TALD (total activity in LD), TADD (total activity in DD), TA (total activity), and PROPDD (proportion of activity in DD). ANOVA with Tukey’s *post-hoc* test and Kruskal-Wallis with Dunn’s multiple comparisons test used when Bartlett test for homoscedasticity was significant. Results of pairwise comparisons for each parameter are shown as superscript letters; in each column the different letters represent significant difference among treatments

Treatment	*n*	TALD	TADD	TA	PROPDD
		Mean ± SD	Mean ± SD	Mean ± SD	Mean ± SD
Males	80	75.83 ± 26.60^ac^	127.60 ± 88.73^a^	203.43 ± 99.21^a^	0.57 ± 0.17^a^
Males_VF	80	84.18 ± 27.68^ab^	176.70 ± 82.64^bcd^	260.88 ± 92.76^bc^	0.65 ± 0.12^bcd^
Males_IF	40	76.86 ± 26.03^ac^	123.31 ± 54.27^ac^	200.18 ± 67.24^a^	0.60 ± 0.13^ac^
Females	80	66.91 ± 27.89^c^	202.09 ± 154.89^d^	269.00 ± 171.34^c^	0.68 ± 0.16^d^
ANOVA *F* (*P*)		5.399 (0.0013)	–	–	–
K-W chi-square (*P*)		–	20.959 (0.0001)	18.062 (0.0004)	26.241 (< 0.0001)

*Abbreviations*: *M_VF* males with virgin females, *M_IF* males with inseminated females

**Fig. 5:**
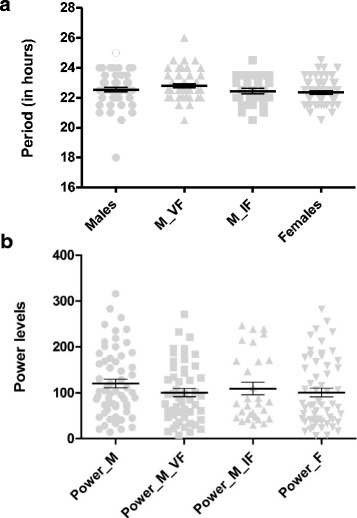
Plot of parameters period and power, calculated when individuals are submitted to constant darkness conditions (see text for details). Mean and standard errors represented by innermost and outer bars, respectively. Comparison among treatments performed by Kruskal-Wallis test, with Dunn’s multiple comparisons test showing no difference among treatments. *Abbreviations*: M_VF, males with virgin females; M_IF, males with inseminated females

The comparison of free-run behavior parameters, period length and power, showed no significant difference among treatments (Fig. 5), which means that the circadian endogenous rhythm is the same regardless of the presence of females and the perception of female’s rhythm in DD. The period length was lower than 24 h for every treatment (~ 23 h, on average), which means that in constant darkness the peak of activity happens earlier each day.

**Fig. 6:**
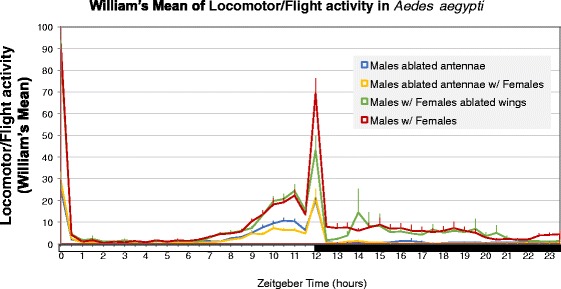
Average profile of the locomotor activity of *Ae. aegypti* males and females with ablated organs, in different treatments, across 24 h (see text for details). X-axis: *Zeitgeber* time refers to the number of h after the light turns on inside the incubator; the white bar represents 12 h of light and the black bar represents 12 h of darkness

**Fig. 7:**
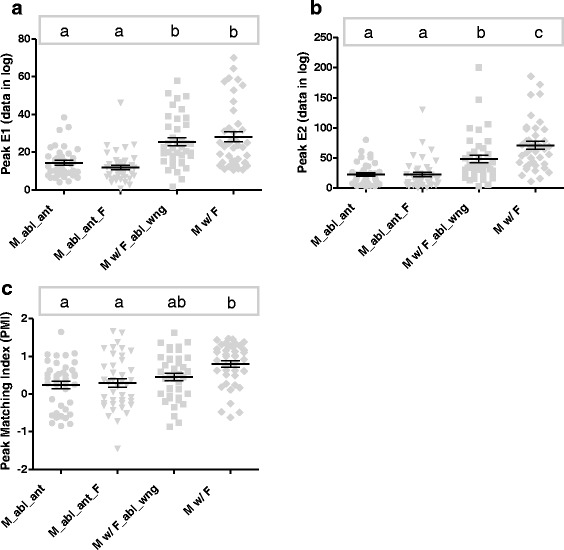
Plot of activity values at evening peaks E1 (**a**) and E2 (**b)** and plot of the peak matching index (PMI) for each treatment (**c**), with means and standard errors represented by innermost and outer bars, respectively. Comparison among treatments performed using ANOVA, with Tukey’s *post-hoc* pairwise tests shown as lowercase letters. *Abbreviations*: M_abl_ant, males with ablated antennae; M_abl_ant_F, males with ablated antennae with females; M w/ F_abl_wng, males with females with ablated wings; M w/ F, males with females

Figure 6 shows the results of Exp5, which used ablated individuals to investigate the signals that might be involved in the communication among males and confined females. The results indicate that males with ablated antennae seem not to change their activity profile when one female is present (*F*_(3, 155)_ = 6.128, *P* < 0.0006, Tukey’s test for PMI: *P* = 0.987) (Table 3), even though the ablated males (148.26 ± 62.42) have a significantly lower total activity (*F*_(3, 156)_ = 66.77, *P* < 0.0001, Tukey’s test: *P* < 0.0001) than not-ablated males (329.72 ± 86.10) (Fig. 6, Table 4).When females with ablated wings are present, the total activity of males is not significantly different from the not-ablated control (*F*_(3, 156)_ = 66.77, *P* < 0.0001, Tukey’s test: *P* = 0.065) (Table 4), although the amplitude of the evening peak E2 is significantly lower (*F*_(3, 155)_ = 25.43, *P* < 0.0001, Tukey’s test: *P* = 0.049) (Fig. 6, Table 3). Females with ablated wings are unable to produce sound, thus males become less active if the females’ wing beats are not noticed. In any case, these males still show a positive value of PMI (0.5 ± 0.6), with peak E2 greater than peak E1, and not significantly different from PMI of males with females not ablated (0.8 ± 0.6) (*F*_(3, 155)_ = 6.128, *P* < 0.0006, Tukey’s test: *P* = 0.082) (Fig. 7c, Table 3), indicating that either chemical or visual cues must be signaling males about a female’s presence.

**Table 3:** Statistical comparisons of locomotor-activity parameters of the ablation experiment: E1 (evening peak of activity 1), E2 (evening peak of activity 2) and PMI (peak matching index). ANOVA with Tukey’s *post-hoc* test used to compare parameters among treatments. Results of pairwise comparisons for each parameter are shown as superscript letters; in each column the different letters represent significant difference among treatments

Treatment	*n*	E1	E2	PMI
		Mean ± SD	Mean ± SD	Mean ± SD
M_abl_ant	40	14.3 ± 7.9^a^	22.6 ± 18.2^a^	0.2 ± 0.6^a^
M_abl_ant_F	40	11.9 ± 7.9^a^	22.8 ± 24.8^a^	0.3 ± 0.7^a^
M w/ F_abl_wng	39	25.5 ± 13.4^b^	48.2 ± 39.4^b^	0.5 ± 0.6^ab^
M w/ F	40	28.1 ± 17.2^b^	71.1 ± 42.0^c^	0.8 ± 0.6^b^
ANOVA *F* (*P*)		19.13 (< 0.0001)	25.43 (< 0.0001)	6.13 (0.0006)

*Abbreviations*: *M_abl_ant* males with ablated antennae, *M_abl_ant_F* males with ablated antennae with females, *M w/ F_abl_wng* males with females with ablated wings, *M w/ F* males with females

**Table 4:** Statistical comparisons of locomotor-activity parameters of the ablation experiment: TALD (total activity in LD), TADD (total activity in DD), TA (total activity), and PROPDD (proportion of activity in DD). ANOVA with Tukey’s *post-hoc* test used to compare parameters among treatments. Results of pairwise comparisons for each parameter are shown as superscript letters; in each column the different letters represent significant difference among treatments

Treatment	*n*	TALD	TADD	TA	PROPDD
		Mean ± SD	Mean ± SD)	Mean ± SD	Mean ± SD
M_abl_ant	40	45.77 ± 17.56^a^	102.48 ± 55.95^a^	148.26 ± 62.42^a^	0.66 ± 0.13^a^
M_abl_ant_F	40	40.45 ± 15.49^a^	97.77 ± 58.59^a^	138.21 ± 65.71^a^	0.67 ± 0.15^a^
M w/ F_abl_wng	40	87.51 ± 27.21^b^	200.28 ± 73.02^b^	287.79 ± 83.65^b^	0.68 ± 0.11^a^
M w/ F	40	101.39 ± 28.24^b^	228.33 ± 75.90^b^	329.72 ± 86.10^b^	0.68 ± 0.08^a^
ANOVA *F* (*P*)		76.61 (< 0.0001)	40.6 (< 0.0001)	66.77 (< 0.0001)	0.186 (0.906)

*Abbreviations*: *M_abl_ant* males with ablated antennae, *M_abl_ant_F* males with ablated antennae with females, *M w/ F_abl_wng* males with females with ablated wings, *M w/ F* males with females

## Discussion

One of the greatest concerns in epidemiology is the expansion of mosquito populations, leading to the spread of mosquito-borne diseases into human populations. Contributing to this expansion are abiotic factors, like the global increase in temperature, favoring the rise of new proper habitats and lengthening the season for the breeding of mosquitoes [56], and the influence artificial light has on the daily activity rhythms of diurnal species [57]. Essentially, the maintenance and spread of mosquito populations is secured by any conditions that lead to individuals’ successful mating, plus significant fecundity and survivorship. Therefore, the mechanisms used by one species for recognizing the proper mating partners represent a primary essential step for its propagation [19].

The collection of behaviors involved in mate recognition and location occurs within a narrow window of time, and characterizing male activity behavior towards finding females of the same species may indicate how behavior can be manipulated for population control. Recent work on acoustic interactions among sexes in *Aedes aegypti* has found that male mating success can be determined by the ability of harmonic convergence, which is itself facilitated by a larger body size [11].

Here we focused on identifying specific parameters of the locomotor activity of *Ae. aegypti* males that may indicate a response to a nearby female. Our design relied on the use of activity monitors to measure any changes in male locomotor/flight activity when a female is present, in relation to the activity of a solitary male. Activity monitors have been used extensively for the study of circadian rhythm in insects, especially in *Drosophila* [48, 58, 59], but also in other dipteran species [49, 50, 55, 60–64]. The advantages of using this system include the possibility of monitoring several individuals at the same time, under the same conditions of photoperiod, temperature and humidity, and the fact that all the data are collected on an automated computer system, eliminating the subjectivity of the observer. Alternative automated methods are usually based on video tracking software, applied to either 2D or 3D videos. While these methods allow the tracking of several individuals simultaneously participating in complex behaviors, which generates activity data that may compare to natural situations of male-male or male-female interactions [65, 66], they use expensive multi-camera gear and rely on computer algorithms to produce flight activity data. The monitoring system used here presents some advantages that fit better to our purposes, for instance: (i) all individuals being monitored in tubes share the same environmental condition (including social environment), meaning that we have replicated activity data to be analyzed with regular statistical packages; and (ii) individuals can be constantly monitored for much longer than videos would allow.

The current work is pioneer in using these monitors to study locomotory/flight activity related with mate recognition and attraction. The adapted confinement tubes allowed us to quantify the response of individual males to nearby females in terms of the extent of activity peak displacement. In this direction, we created the peak matching index (PMI), a new parameter that we anticipate could be used to measure the inclination of males to respond to females’ presence, and hence, tested as an indicator of mating success. If the window of pre-mating male behavior relies on species-specific recognition, we predict that PMI would be significantly different between males exposed to same-species females and males exposed to different-species females. Interestingly, we did not find significant difference in PMI between males with virgin females and males with inseminated females (Fig. 3), even though the total activity (LD 12 h/12 h + DD) was significantly greater in males with virgin females. This indicates that males switch their peak of activity regardless of female’s condition, but may respond with lower activity if inseminated females are themselves less active. Alternatively, it is possible that males only recognize that a female is inseminated when the female refracts to the mating attempt, suggesting that males’ response in the pre-encounter and in the encounter are decoupled.

The antennae of mosquito males are among the most powerful hearing organs in animals, indicating that sound is the main cue involved in recognition of females of the same species [4, 28, 30, 31]. Wishart & Riordan [67] argued that males of *Ae. aegypti* are capable of detecting the sound of a conspecific female even when the background noise is 10 times louder. Although males of *Ae. aegypti* are described as opportunistic because they can mate at any time they find a female in flight [26], the fact that resting males are not promptly stimulated to flight by a flying female nearby [27, 67] suggests that either the female’s wing beats are not the main cue for males, or a male must be in flight in order to find a stimulus to chase flying females. In the species *C. quinquefasciatus*, a recent investigation on the harmonic convergence between flight tones of males and females has shown that males rely on their own flight tone oscillation to recognize, locate and position themselves towards flying females [68], implying that males must be flying to initiate the progression towards mating success.

Our results challenge the view that the swarming behavior is necessary for a male’s response to a female’s presence, as a single male in the glass tube shows to be responsive to a female’s cues. Using the same adapted monitoring system, we explored organ-ablation in males and females as a way of characterizing alterations in the locomotor activity profile that could possibly reveal the nature of the females’ signals noticed by males. These experiments showed that a male’s response can be seen even when females are wing-ablated and unable to produce sound, indicating that visual or chemical cues might play a role.

Although we do not have a clear-cut answer for which females’ signals the males are reacting to, our results with organ-ablated mosquitoes suggest that males’ antennae are essential for proper flight activity and significant female recognition (Fig. 6). This was expected, since the male antennae are the organs that capture both acoustic and chemical signals [25], but may also work on securing proper balance when the individual is flying, which might explain why the total activity is much lower in antennae-ablated males (Figs. 6, 7). The ablation of females’ wings caused a reduction in males’ activity, but note that this happened uniquely in the second peak of males’ activity, which is the peak we attribute to males’ response to the presence of females. This suggests that acoustic signals (female’s wing-beats) stimulate an important portion of males’ response, but chemical signals like volatile pheromones might be playing a role in causing some of males’ response.

Genetically-modified mosquitoes have been a promising weapon for reducing vector population growth [3, 17, 69, 70]. However, the release of millions of mosquito males has been done without testing these males for their potential of mate recognition as a first step towards reproductive success [3, 10, 11]. As a future purpose, we suggest that the adapted monitor system described here could be tested as a possible resource to associate the male’s response to females to its efficiency in mating. If this association is confirmed, the adapted monitor system could be used for screening modified males for their capacity of outcompeting field males on the recognition of females and mating.

## Conclusions

The study of male reproductive behavior in mosquitoes *Aedes aegypti* has the potential for contributing to the design of new strategies for tackling the growth of their populations. Here we used an innovative adapted monitoring system to focus on the male’s locomotor/flight activity in response to females. We found that males are able to significantly change their activity profile when they notice the presence of one female. Furthermore, we found that the females’ wing beats are not the only cues noticed by males; chemical and/or visual signals may also be in play to provoke the switch in the activity peak. We propose that the ability of reacting to these signals could be tested as a potential indicator of one male’s success in finding a female and mating. Testing this hypothesis is a future purpose of great relevance, since it may help creating a method to evaluate pre-mating activity of genetically modified mosquitoes.

## Abbreviations

ANOVA: Analysis of variance
DD: Dark/dark
E1: First evening peak
E2: Second evening peak
LD: Light/dark
M w/ F: Males with females
M w/ F_abl_wng: Males with females with ablated wings
M_abl_ant: Males with ablated antennae
M_abl_ant_F: Males with ablated antennae with females
M_IF: Males with inseminated females
M_VF: Males with virgin females
PMI: Peak matching index
ZT: *Zeitgeber*
